# Continuous false positive results by SARS-CoV-2 rapid antigen testing: a case report

**DOI:** 10.3389/fpubh.2023.1240308

**Published:** 2023-11-03

**Authors:** Yannick Galipeau, Abishek Xavier, Aaron Dyks, Curtis Cooper, Marc-André Langlois

**Affiliations:** ^1^Department of Biochemistry, Microbiology & Immunology, Faculty of Medicine, University of Ottawa, Ottawa, ON, Canada; ^2^The Ottawa Hospital Research Institute, Ottawa, ON, Canada; ^3^University of Ottawa Centre for Infection, Immunity and Inflammation (CI3), Ottawa, ON, Canada

**Keywords:** SARS-CoV-2, rapid antigen test (RAT), false positive, case report, COVID-19

## Abstract

Efficient and rapid identification of active SARS-CoV-2 infections has been key to monitoring and mitigating the spread of the virus. The implementation of nucleic acid testing (e.g., RT-PCR) was broadly adopted by most public health organizations at the national and community levels across the globe, which was followed by more accessible means of home testing including lateral flow immunochromatographic assay (LFA), also known as a rapid antigen test. Here we report the case of an adult female who repeatedly and consecutively tested positive by RAT (BTNX inc). This sustained false positive was not linked with an active SARS-CoV-2 infection, which was ruled out by RT-PCR and serological analyses. SARS-CoV-2 serology revealed no detectable levels of antibodies against the nucleocapsid suggesting no recent prior infection by SARS-CoV-2. This continuous false positive was limited to BTNX testing devices. This case report aims to describe that such continuous false positives can occur and describes alternative testing approaches that can be performed to confirm RAT results. In addition, broader awareness of such occurrences is warranted in the healthcare and public health community to avoid unnecessary negative impacts on individual’s day to day life.

## Introduction

The ability to rapidly identify SARS-CoV-2 infected individuals has been central to most public health measures across the globe and is a major component to contact tracing initiatives. Population-level testing requirements prompted the establishment of testing centers that conducted viral nucleic acid detection (i.e., RT-PCR) (1, 2). Despite being the gold standard detection method of SARS-CoV-2 in clinical samples, and its broad use early in the pandemic, RT-PCR testing has some important limitations (i.e., expensive laboratory equipment, reliance on highly qualified laboratory and medical workers, non-point-of-care testing, etc.). In light of these limitations, countries like Canada have endorsed the usage of at-home Rapid Antigen Tests (RAT) (3–5).

Several studies have investigated RAT performances in real life settings. In a cohort of 72,382 paired RT-PCR and RAT, Eyre et al. reported an overall sensitivity of 63.2% (6). This estimate is in line with other studies (7, 8). While sensitivity is increased in symptomatic individuals, RATs overall have a lower sensitivity compared to RT-PCR. In addition, despite concerns that SARS-CoV-2 RAT could cross-react with other pathogens such as seasonal coronaviruses (OC43, 229E, NL63, HKU-1), RATs are regarded as highly specific. In fact, several studies reported specificities that ranged from 99.71% (6), 99.95% (9) 100% (10).

Given that a vast array of RATs have been authorized by the federal regulatory bodies, their relative real-world performance, technology used, and their usage vary (10, 11). In this case report, we will focus primarily on the RAT from BTNX inc (COV-19C25). This lateral immunochromatographic at-home test kit is designed to detect SARS-CoV-2 nucleocapsid in nasopharyngeal secretions (12). Here we report the case of a 47 year old female who consistently and consecutively tested positive by RAT despite any laboratory or clinical evidence of SARS-CoV-2 infection.

### Case description

A 47-year-old Caucasian female first tested positive by rapid antigen test (RAT) on December 28th 2021. At that time, she remained positive for over 2 weeks until she stopped self-testing. Following Canadian public health recommendations, she tested herself regularly by RAT after a high-risk contact, or when experiencing COVID-19 associated symptoms. Repeated self-testing with RAT yielded a constant positive result. After each positive RAT result, she followed recommendations from public health to self-isolate. She was careful about masking and wore N95 in public spaces. Given that RATs were repeatedly positive, she contacted public health authorities for guidance and they recommended she stop testing. On the other hand, her family physician told her to keep testing, thereby suggesting that she was still contagious. These conflicting recommendations have led to confusion, distress and uncertainty for the subject. The numerous isolation periods have impacted her day-to-day life, impacted her family, and prevented her from visiting friends and relatives.

We were contacted in early fall 2022 to offer guidance on the continuous SARS-CoV-2 positive results on RAT given the mixed recommendations from her physician and local public health. Her medical history included an allergy to dust mites and mild asthma that did not require medication. Her SARS-CoV-2 vaccination history included a first dose of (Pfizer) on May 1st 2021, a second dose of (Moderna) on June 19th 2021, a third dose of (Moderna) on November 19^th^ 2021, and a fourth dose of (Pfizer) on August 9th 2022.

**Figure 1:**
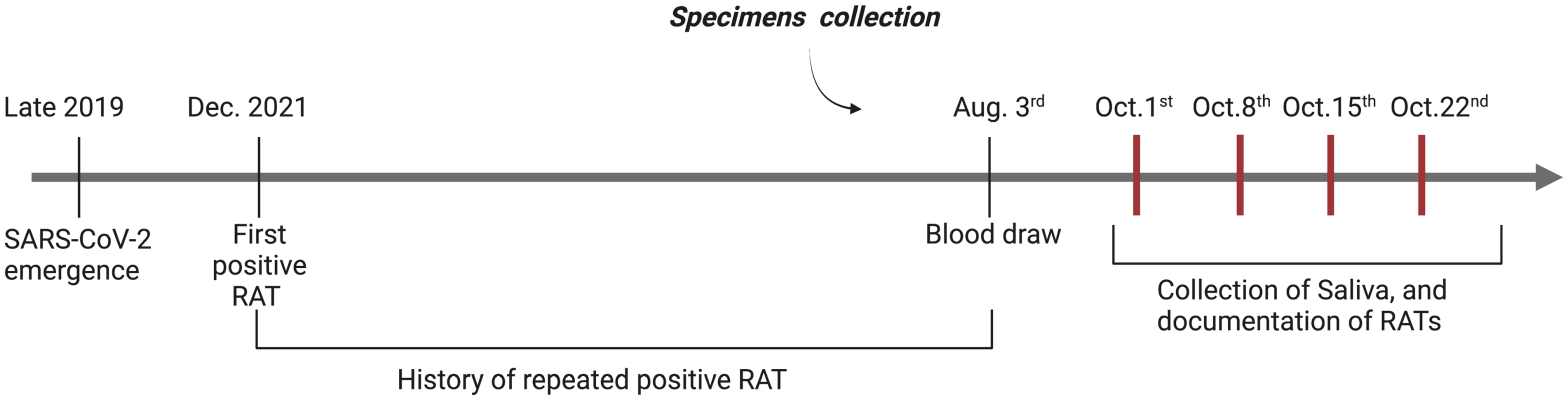
Timeline overview of positive RATs and specimen collection. A visual representation of both the subject testimony in regards to the history of positive RATs and sample collection (Blood, RATs) as part of this case report. Red vertical bars represent the four weeks where we documented positive RAT for this case report, and where the subject provided saliva samples.

We collected a blood sample to investigate SARS-CoV-2 associated antibodies in serum. We also requested that she provide a saliva sample every week for a month for molecular testing. At the time of providing the saliva sample, we also asked that she perform a set of RAT tests, which included 3 BTNX tests. One test was performed following the manufacturer’s instructions (swabbing of the nasal cavity), the other was a swab of the oral cavity, and the third comprised of combination swabbing techniques of both sites. For the first week, a RAT from another supplier was also performed simultaneously (Figure 1).

**Figure 2:**
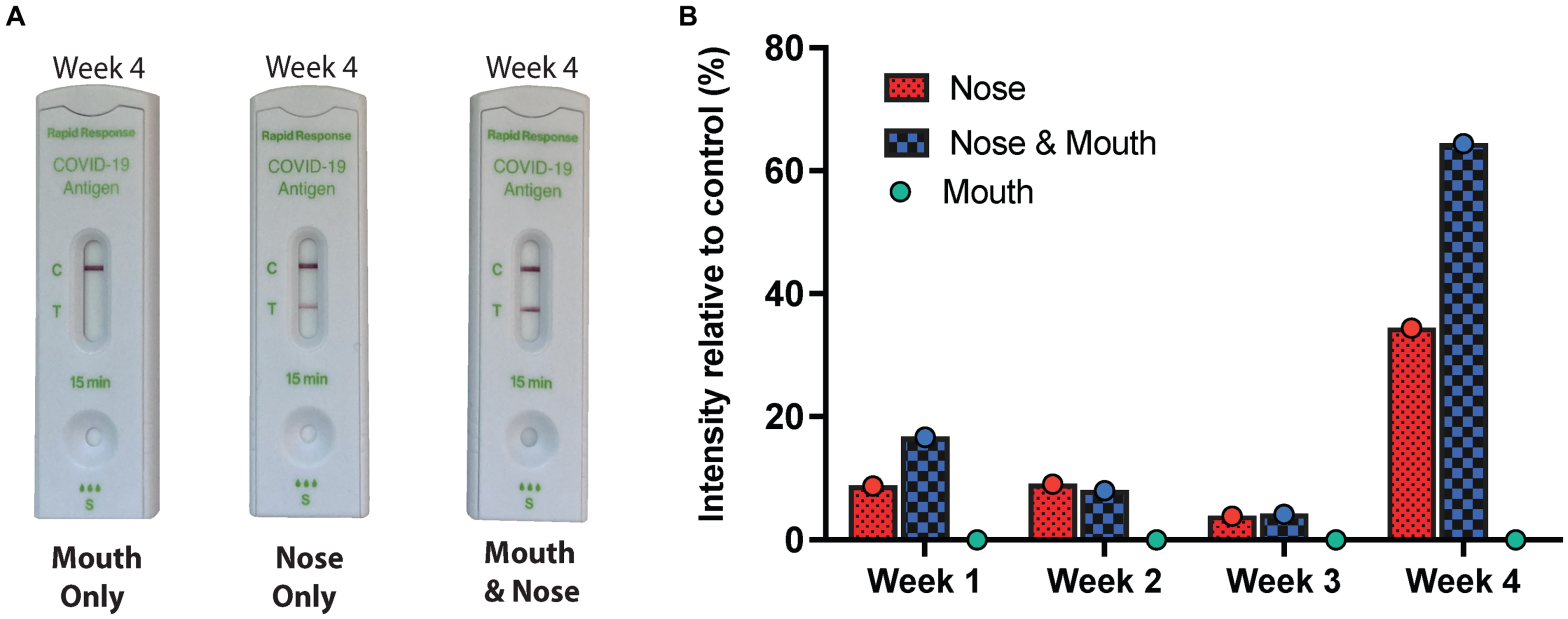
Weekly results of RAT by swabbing technique and location. **(A)** An overview of the RATs by swabbing technique for week 4 from photographed RAT performed at home. As described, the nasal cavity was swabbed (deep and anterior nasal swab), the oral cavity (throat and cheeks) as well as the combination of both swabbing techniques. The RATs were performed sequentially in the same session and photographed. **(B)** An overview of the RAT’s band intensity in relation to the control band for every week and for the three swabbing techniques.

**Figure 3:**
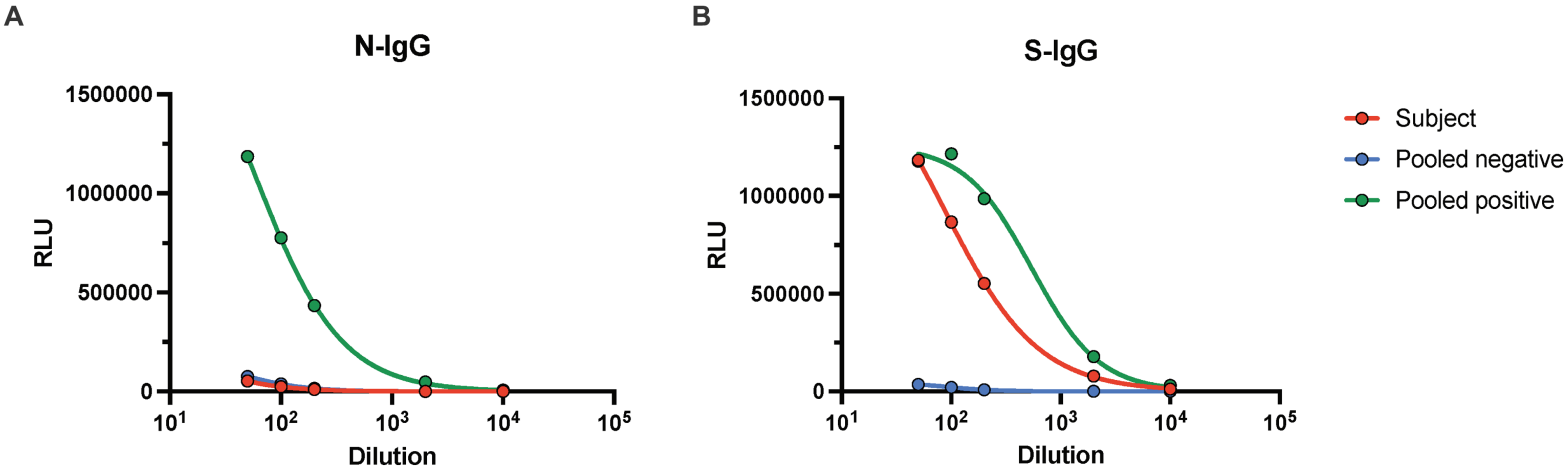
Serology results of serum sample provided prior to week 1. A blood sample was provided prior to performing the RATs, and serum was isolated. Antibodies against the SARS-CoV-2 **(A)** nucleocapsid and **(B)** Spike protein was performed using a previously established chemiluminescent ELISA serological assay. IgG antibodies were measured by titrating the subject’s serum sample (red). In addition, a titration of a negative (blue) and positive (green) pooled serum from residual blood was included as positive and negative controls. Here the blank adjusted relative luminescence units (RLU) intensity is shown. IgG antibody titer against the nucleocapsid was below conversion threshold, IgG titer against the spike protein was estimated to be 130.41 BAU/mL.

All four weekly RATs that were performed following manufacturer recommended swabbing techniques or the combination of oral and nasal swabbing techniques yielded unambiguous positive results. All four weekly RATs performed by oral swabbing were negative (Figure 2). Interestingly, a RAT from another supplier (FlowFlex) was performed in the first week and was negative, suggesting that the positivity might be limited to BTNX kits only. Nucleic acid testing from the saliva samples provided for each week was negative for SARS-CoV-2 E and N gene, thus suggesting the absence of an active or persistent infection. SARS-CoV-2 IgG antibody measurements against the N and S proteins were performed. Only antibodies against the Spike protein were detected beyond our positivity threshold (Figure 3). Given the lack of antibodies against the N protein, a recent SARS-CoV-2 infection was deemed unlikely.

## Discussion

While false positives occur with any diagnostic tests, including RATs, here we report the case of a continuous false positive. To document the events related to the positive RAT and to investigate potential sources of cross-reactivity, a series of RATs were performed. One weekly RAT was performed for four weeks by anterior and deep nasal swabbing as per manufacturer’s instructions. Each of these RATs were positive, confirming the subject’s testimony (Figure 2B). Given that Canadian public health authorities were also recommending individuals to swab the oral cavity (throat and cheeks) in combination with anterior and deep nasal swabbing to increase sensitivity (13, 14), we also asked that a RAT with both swabbing techniques (oral and nasal swabbing) and oral swabbing alone be performed sequentially in one session. Interestingly, all of the oral cavity RATs were negative (Figure 2). The RATs performed with the combinatorial swabbing technique echoed the results seen with the nasal cavity swabbing technique and were all positive. Notably, the signal intensity of the RATs on week four was higher than previous three weeks. Given that RATs are non-quantitative tests it is difficult to comment if this is caused by a better swabbing technique resulting in a greater amount of biological material applied to the RAT or a true increase in signal. In addition, a RAT from another supplier was performed in parallel on week one and resulted in a negative result. This echoes previous documented RATs from the participant outside of this study’s timeframe where RAT from FlowFlex and Abbott remained negative despite a concurrent positive on BTNX RAT.

While the subject did not display any COVID-19 related symptoms at the time of taking the RATs, we still collected saliva samples to perform nucleic acid testing given that subclinical asymptomatic SARS-CoV-2 infections have been documented (15–18). The multiplex qPCR showed no detectable levels of SARS-CoV-2 N and E genes. An internal control (Human RPP30 gene) was also included to validate RNA extraction efficiency from the saliva. These data also align with the fact that no close contacts to the subject tested positive or developed COVID-19 related symptoms. In light of this, the possibility of an active SARS-CoV-2 infection was ruled out.

SARS-CoV-2 antigen deposition in various tissue following infection have been documented previously (19, 20). As such, we next investigated whether the positive RAT could be from deposited nucleocapsid protein from a prior infection. We measured IgG antibodies against the spike protein and the nucleocapsid of SARS-CoV-2 (Wuhan). We calculated an overall titer of 130.41 BAU/mL of anti-spike IgG antibodies, while antibodies against the N protein were below the seroprevalence threshold. Given the lack of antibodies recognizing the nucleocapsid, we can conclude that the spike antibodies are vaccine-elicited and that prior to performing the RAT, this subject had no serological markers suggesting a prior infection. While it is true that antibodies decay with time, it is unlikely that a RAT would detect true nucleocapsid protein without an associated detectable antibody response and/or a positive RT-PCR result.

The source driving the false positive signal on BTNX devices remains unclear. In principle, endogenous peptides could cross-react with BTNX RAT antibodies within the devices. It is also possible that cross-reactivity be driven by exogenous peptides from the microbiome. Given the proprietary nature of the antibodies used in each RATs by the various manufacturers, further investigation on how the antibody selection contributes to false positives is difficult. Anecdotally, the subject’s children and family direct members did not generate a false positive result on RATs.

## Conclusion

Continuous false positives can occur with RATs despite their overall high positive predictive value. A confirmatory RAT from another supplier could be used as confirmation. Molecular testing remains the gold standard to confirm active infections. Serological testing can rule out chronic or previous SARS-CoV-2 infections in the vast majority of cases. Increased awareness by the medical and public health community of the existence of individuals that continuously test positive is warranted to avoid unnecessary isolation periods and subsequent impact on an individual’s mental health, family, professional activities, social and day-to-day life.

Detailed descriptions of the methods are available as Supplementary material.

## Data availability statement

The raw data supporting the conclusions of this article will be made available by the authors, without undue reservation.

## Ethics statement

Ethical approval was obtained from the University of Ottawa REB (H-11-20-6172) for the studies involving humans. The studies were conducted in accordance with the local legislation and institutional requirements. The participants provided their written informed consent to participate in this study. Written informed consent was obtained from the individual(s) for the publication of any potentially identifiable images or data included in this article.

## Author contributions

YG and M-AL wrote the manuscript. YG performed the serology experiments and created the figures. AX performed the qPCR experiments. AD provided participant coordination and sample collection. CC offered clinical perspective and insights. All authors contributed to the article and approved the submitted version.
