# Supplementary material for: Continuous false positive results by SARS-CoV-2 rapid antigen testing: a case report
Source: Front Public Health. 2023 Nov 3;11:1240308. doi: 10.3389/fpubh.2023.1240308 (PMC10657208; doi:10.3389/fpubh.2023.1240308)
Supplement: Supplementary file 1 [file Table_1.docx]

**SUPPLEMENTARY MATERIAL**

**Methods**

*Sample Collection*

Blood was collected using an SST vacutainer and serum was isolated after a 1300xg spin at 4°C for 5 minutes. The specimen was subsequently stored at 4°C until antibody levels were measured.

*Measurements of antibody levels in sera*

Antibodies were measured using a high-throughput direct chemiluminescent ELISA (full methods described previously) (1). Briefly, SARS-CoV-2 antigens (spike ectodomain (Wuhan) with S1/S2 furin site mutations, prefusion stabilizing mutations and human resistin as a trimerization partner and nucleocapsid (amino acids 1–419 of YP_009724397)) were generously provided by Dr. Yves Durocher, National Research Council of Canada (NRC), Montréal (2). The assays were performed manually, in a 384 well format. Initially, 50ng /well of the antigen of interest diluted in PBS was coated in a high-binding polystyrene Nunc plate (Thermo Fisher Scientific, #460372) in a final volume of 10uL. The plates were then centrifuged at 216xg and incubated overnight at 4°C and then washed four times with 100uL of PBS-T. The plates were then blocked with 80uL of 3% w/v skim milk powder dissolved in PBST for 1 h and then washed (as described before). Samples or controls were diluted to the indicated dilution in 1% w/v skim milk powder dissolved in PBST and 10uL was added to the respective wells. Controls included a calibration curve anti-SARS-CoV-2 S CR3022 Human IgG1 (Absolute Antibody, Ab01680-10.0), Human anti-Nucleocapsid IgG (Genscript -HC2003 A02039)pooled human sera, and non-specific Ig control. After a 2h incubation, the plates were washed once more and 10uL of the secondary antibody was added to each well and incubated for another hour. Secondary antibodies used were anti-Human IgG#5-HRP (Dr. Yves Durocher, NRC) diluted 1 in 5400. After the incubation, plates were washed one last time and 10uL of ELISA Pico Chemiluminescent Substrate (Thermo Fisher Scientific #37069) diluted 1:2 in ddH_2_O was added to each well and incubated for 5 minutes. Luminescent signal was measured on a Neo2 plate reader (Biotek) at 20ms/well at a read height of 1.0mm.

An antibody titer in international units (BAU/mL) was calculated as previously described (1). Briefly, the WHO international standard (20/136) was titrated and modelled using a four-parameter log-logistic regression with the DRC package in R. Blank adjusted luminescence values of the samples were scaled by dividing the values by the inflection point of the isotype-antigen specific calibration curve present in quadruplicate on each plate. Scaled luminescence values in the linear range were then converted to BAU/mL.

*Measurement of Viral RNA in saliva*

Saliva was collected using a saliva collection tube (DNA Genotek: OMNIgene·ORAL OM- 505) in the morning, prior to food or liquid intake. Tubes were kept at room temperature until they were processed for vRNA extraction using the QIA amp Viral RNA Mini Kit (Qiagen, Cat#52906) following the manufacturer’s instructions with minor modifications. Prior to RNA extraction, all samples were incubated in a water bath at 50°C for 30 min prior to RNA extraction. 140µL of each sample was used for extraction in duplicate and the RNA was eluted with 60µL of AVE buffer. qPCR was performed using Reliance One-Step RT-qPCR Kit (Bio-Rad #12010176). Reactions were set up with 10 µL of RNA, 5 µL 4X Reliance RT-qPCR supermix, 1µL 20x primer/probe), and 4 µL of nuclease free water to a final reaction volume of 20 µL. A list of primers and probes used can be found below. A CFX96 Touch^TM^ Real-Time PCR (Bio-Rad) instrument was used with the following conditions initial RT at 50°C, 60 minutes, followed by polymerase activation at 95°C for 10 minutes, and 40 cycles of denaturation, annealing/extension at 95°C/10 s, then 55°C/60 s, respectively.

**N gene :**

Probe : 5FAM/ACCCCGCAT/ZEN/TACGTTTGGTGGACC/3IABkFQ

Primer FWD : GACCCCAAAATCAGCGAAAT

Primer REV : TCTGGTTACTGCCAGTTGAATCTG

**E gene :**

Probe : 5TexRd-XN/ACACTAGCCATCCTTACTGCGCTTCG/3IAbRQSp

Primer FWD : ACAGGTACGTTAATAGTTAATAGCGT

Primer REV : ATATTGCAGCAGTACGCACACA

**Human RPP30 :**

Probe : 5HEX/TTCTGACCT/ZEN/GAAGGCTCTGCGCG/3IABkFQ

Primer FWD : AGATTTGGACCTGCGAGCG

Primer REV : GAGCGGCTGTCTCCACAAGT

*Rapid antigen tests*

A series of RATs was performed in parallel for 4 weeks. These included one RAT from BTNX (COV-19C25) performed in accordance with the manufacturer’s instructions (anterior and deep nasal swabbing), one RAT from BTNX (COV-19C25) performed by swabbing the oral cavity (throat and cheeks), and one RAT from BTNX (COV-19C25) performed with a combination or nasal and oral swabbing. BTNX RATs used in the timeframe of this study had the following lot numbers: I2203054, I2202101, I2202287, I2201363. In addition, for the first week, an additional RAT from FlowFlex (L631-118B5) was performed following the manufacturer’s recommendations. ImageJ was used to quantify band intensity from the RATs photos : (band intensity / Control band intensity) x100% (3)

References

1. Colwill K, Galipeau Y, Stuible M, Gervais C, Arnold C, Rathod B, et al. A scalable serology solution for profiling humoral immune responses to SARS-CoV-2 infection and vaccination. Clinical & Translational Immunology. 2022;11(3):e1380.

2. Stuible M, Gervais C, Lord-Dufour S, Perret S, L'Abbé D, Schrag J, et al. Rapid, high-yield production of full-length SARS-CoV-2 spike ectodomain by transient gene expression in CHO cells. J Biotechnol. 2021;326:21-7.

3. Schneider CA, Rasband WS, Eliceiri KW. NIH Image to ImageJ: 25 years of image analysis. Nature Methods. 2012;9(7):671-5.
